# A new small supernumerary marker chromosome, generating mosaic pure trisomy 16q11.1–q12.1 in a healthy man

**DOI:** 10.1186/1755-8166-1-4

**Published:** 2008-04-02

**Authors:** Laura Rodríguez, Tomas Liehr, María Luisa Martínez-Fernández, Ana Lara, Antonio Torres, María Luisa Martínez-Frías

**Affiliations:** 1 Estudio Colaborativo Español de Malformaciones Congénitas (ECEMC) del Centro de Investigación sobre Anomalías Congénitas (CIAC), Instituto de Salud Carlos III, Ministerio de Sanidad y Consumo, Madrid, Spain; 2 Center for Biomedical Research on Rare Diseases (CIBERER), Madrid, Spain; 3 Institut für Humangenetik und Anthropologie, Friedrich Schiller Universität Jena, Germany; 4 Servicio de Pediatría, Hospital San Juan de la Cruz, Úbeda, Jaén, Spain; 5 Departamento de Farmacología, Facultad de Medicina, Universidad Complutense, Madrid, Spain

## Abstract

Here we report on a healthy and fertile 30 years old man, who was carrier of a small supernumerary marker chromosome (sSMC). The application of molecular techniques such as fluorescence *in situ *hybridisation (FISH), microdissection and reverse painting, helped to characterize the sSMC which resulted to be derived from chromosome 16. In fact, the presence of euchromatin material from the long arm (16q) in the sSMC was demonstrated, and the karyotype can be written as mos 47, XY,+min(16)(:p11.1->q12.1:)[20]/46, XY [10].

## Brief report

Small supernumerary marker chromosomes (sSMC) have been recently defined by Liehr et al. [1] as "structurally abnormal chromosomes that cannot be identified or characterized unambiguously by conventional banding cytogenetics alone, and generally are equal in size or smaller than a chromosome 20 of the same metaphase spread". These sSMC have been described from all human chromosomes although most of them are derivatives of acrocentric chromosomes [1,2], and approximately 30% of them were parentally inherited [3,4].

The individuals carrying a sSMC, have a wide range of clinical variability, which may be related with the different sizes of the sSMC, the presence and/or absence of euchromatic material, the degree of mosaicism and/or uniparental disomy (UPD) [2]. Indeed, 70% of non-acrocentric sSMC do not have phenotypic repercussion, while the remaining 30% have different clinical manifestations [5]. Therefore, it is very important to characterize the content and the structure of the sSMC, in order to establish an adequate genotype-phenotype correlation.

**Figure 1:**
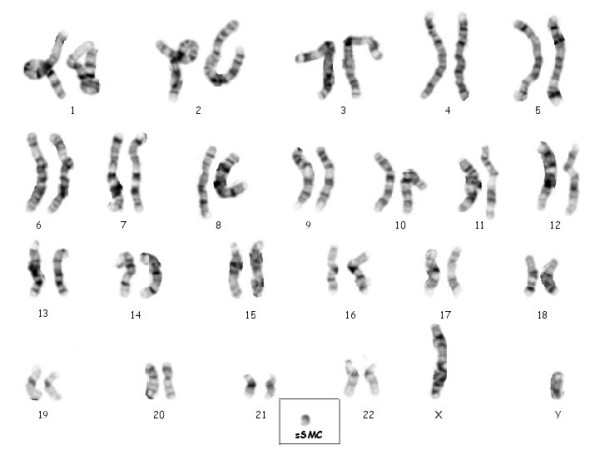
High resolution G-band chromosomes karyotype showing the sSMC.

**Figure 2:**
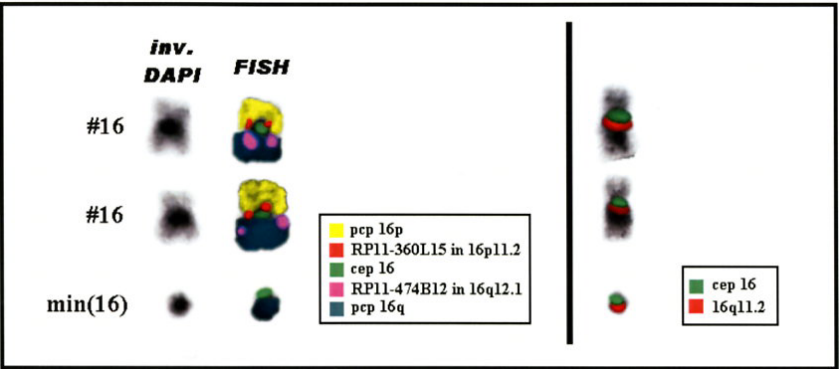
Centromere-specific multicolour fluorescence in situ hybridization (FISH) and subcentromere specific multicolor FISH (subcenM-FISH) showing that the sSMC was from a chromosome 16 origin, containing centromeric, heterochromatic and euchromatic material from the long arm of chromosome 16 (16q).

The present possibilities of the new molecular techniques such as fluorescence *in situ *hybridisation (FISH), microdissection and reverse painting, are enhancing our knowledge about sSMC. Here we present the result of using these techniques to characterize a sSMC, which was found in a blood sample of a healthy male. He is a healthy and fertile 30 years old man, father of a malformed newborn (NB) infant who was cytogenetically studied according to standard procedures, because of dysmorphic features at birth and resulted to have on her blood sample high resolution G-band karyotype, a tiny interstitial deletion on a chromosome 2 [46, XX, del(2)(q36.3)] (no other tissues were studied on this patient). A high resolution G-band karyotype was performed on her parents blood samples, and the 2q chromosome deletion of the NB was diagnosed to be "de novo". Nevertheless, unexpectedly, the father karyotype showed two different cellular lines. The first one, with 47 chromosomes was found in 20 cells (66.6%) having a sSMC (Fig. 1), and the second one with 46 chromosomes was observed in the 33.3% (10 cells). The application of centromere-specific multicolour fluorescence *in situ *hybridization (FISH) together with subcentromere specific multicolor FISH (subcenM-FISH) [6] and glass-needle based microdissection [7], revealed that the sSMC was from a chromosome 16 origin, containing not only centromeric and heterochromatic material but also euchromatin from the long arm of chromosome 16 (16q)(Fig. 2). Consequently, his karyotype resulted to be: mos 47, XY, +min(16)(:p11.1->q12.1:)[20]/46, XY[10]. This alteration was produced "de novo" since his parents blood karyotypes were normal, and seems to have no relation with her daughter chromosome interstitial deletion, which was also produced "de novo". An EBV-transformed, immortalized cell line has been established and included in a cell bank [8].

The chromosome 16 long arm (16q) is characterized by the presence of a block of heterochromatin located between its centromere and the euchromatin region. Trisomy of the heterochromatin block is not related with any clinical manifestations, in fact there are five reported cases with sSMC (16), whose content is limited to the centromeric D16Z and heterochromatin regions without clinical manifestations. Among these five cases, the first one, had an abnormal phenotype attributed to an obstetric trauma [9]. The second case ended in a termination of pregnancy (TOP) with no clinical information [10]. And finally, the other three cases were ascertained in the amniotic fluid of three pregnant woman being normal the prenatal outcome and/or baby follow-up [11-13].

Trisomy of the next euchromatic region of the long arm of chromosome 16 (involving the q12 band) has been reported in seven case reports and seven members of two families [14-20] and after a recent review of the clinical data from all those patients, Barber et al. [20] concluded that "duplications of proximal 16q do not have characteristic facies but frequently have short stature, developmental delay, speech delay, learning difficulties and behavioural problems which range from mild to severe". A few genes has been located at 16q12-13 band, such as the Hereditary cylindromatosis gene (*CYLD1*), which has been defined as a tumor suppressor gene [21], the beta subunit gene of the Phosphorylase kinase (*PHKB*) which is an enzyme that activates glycogen phosphorylases in muscle, liver, and other tissues [22] and the human smooth muscle myosin heavy chain (MHC) which corresponds to *MYH11 *gene, and is expressed in the human umbilical artery, bladder, esophagus and trachea [23].

The case we present here, has a partial trisomy of the long arm of chromosome 16, involving the heterochromatin block and the proximal euchromatin region at 16q12 band, and as far as we know is the first one reported in a phenotypically normal patient, with no developmental delay neither mental retardation. It was produced "de novo", and consequently we could not disregard that it could be due to low repetitive elements present in the pericentromeric region of chromosome 16, as recently proposed by different authors for chromosomes 2, 10 and 12 [24-26]. And it was found as a mosaicism, although other tissues were not studied, where different degrees of mosaicism may be present influencing the patient phenotype.

In conclusion, the advantage of molecular techniques have allowed to know the real euchromatic/heterochromatic content of the sSMC(16) in our patient. In fact, they helped to show that not all the euchromatic trisomies are associated with clinical repercussion, as previously reported by Barber [27]. Nevertheless, more affected and non-affected patients are needed to be described in order to elucidate the phenotype-genotype correlations of different sSMC, that we consider is essential for a right genetic prenatal counselling.

## Authors' contributions

LR, carried out the high resolution G-band cytogenetic studies from the patient and his family as well as the preliminary FISH techniques and drafted the manuscript. TL carried out the application of centromere-specific multicolour FISH, the subcentromere specific multicolor FISH (subcenM-FISH) [6] and glass-needle based microdissection techniques. MLMF, help with the high resolution G-band cytogenetic studies. AL and AT are the pediatricians who follow the child and sent us the clinical data and the blood samples to perform the citogenetic and molecular studies. MLMartínez-Frías participated in the design of the manuscript. All the authors read and approved the final manuscript.
